# Rapid detection of *Staphylococcus aureus* in blood culture samples using human IgG-based lateral flow assay

**DOI:** 10.1128/spectrum.03046-23

**Published:** 2024-01-17

**Authors:** Arpasiri Srisrattakarn, Nicha Charoensri, Jeerati Prompipak, Wajeeorn Ouancharee, Bhanubong Saiboonjan, Patcharaporn Tippayawat, Aroonwadee Chanawong, Lumyai Wonglakorn, Ekgarak Kanwattanee, Sirikan Piyapatthanakul, Thitimar Masmalai, Anisara Ariyapim, Rinjong Promson Kendal, Aroonlug Lulitanond

**Affiliations:** 1 Centre for Research and Development of Medical Diagnostic Laboratories, Faculty of Associated Medical Sciences, Khon Kaen University, Khon Kaen, Thailand; 2 Center for Innovation and Standard for Medical Technology and Physical Therapy, Faculty of Associated Medical Sciences, Khon Kaen University, Khon Kaen, Thailand; 3 Clinical Microbiology Unit, Srinagarind Hospital, Khon Kaen University, Khon Kaen, Thailand; 4 Clinical Microbiology Laboratory, The Queen Sirikit National Institute of Child Health, Bangkok, Thailand; 5 Clinical Laboratory, Queen Sirikit Heart Center of the Northeast, Khon Kaen University, Khon Kaen, Thailand; 6 Khon Kaen Hospital, Khon Kaen, Thailand; Johns Hopkins Hospital, Baltimore, Maryland, USA

**Keywords:** lateral flow immunoassay, positive blood culture samples, rapid detection, *Staphylococcus aureus*

## Abstract

*Staphylococcus aureus* is one of the most common pathogens. The conventional workflow for identifying this organism is time-consuming and takes up to several days. Therefore, we developed a colloidal gold-based lateral flow immunoassay (LFIA) using human IgG as a conjugated antibody to detect *S. aureus*. One hundred and thirty-eight clinical isolates, including 79 *S*. *aureus* and 59 non-*S*. *aureus* were spiked in blood samples, and incubated at 37°C for 24 h. The bacterial antigens were simply extracted before being tested by the developed LFIA strips. The results were read by the naked eye within 15 min. Conventional PCR was used as a reference method. The sensitivity and specificity of the developed LFIA were 100% (95% CI: 94.2%–100.0% and 92.4%–100.0%, respectively) in spiked blood culture samples. The detection limits of the LFIA for the purified protein A and bacterial colonies were 10^−3^ µg/mL and 10^7^ CFU/mL, respectively. The performance of the LFIA testing in 221 bacterial colony isolates and 118 positive blood culture bottles from three hospitals by their medical technologists showed 98.1% (95% CI: 94.1%–99.5%) and 89.7% (95% CI: 79.3%–95.4%) sensitivity, respectively. The LFIA is a quick, easy, and sensitive method for detecting *S. aureus* without expensive equipment. It might have the potential for early diagnosis of routine service in low-resource laboratories, leading to a rapid and effective treatment.

**IMPORTANCE:**

In this study, we modified our previously developed lateral flow immunoassay (LFIA) test for the detection of *Staphylococcus aureus* by using an in-house human IgG as a conjugated antibody instead of the specific commercial antibody. It gave comparable results to the former developed-LFIA test and helped cost reduction.

## INTRODUCTION

*Staphylococcus aureus* is an important bacterial pathogen in humans, causing various critical infectious diseases such as septicemia, toxic shock syndrome, pneumonia, and infective endocarditis (1, 2). *S. aureus* is one of the leading pathogens of bloodstream infections, the second rank to *Escherichia coli*. The hospital mortality rate for *S. aureus* bacteremia ranges from 15% to 40% (3). Delays in identifying sepsis increase mortality in advanced stages of severity or septic shock (4). Therefore, rapid identification and early treatment further decreased the number of severe cases and yearly deaths.

Various approaches for early identification of bacteria from a positive blood culture include Gram staining, conventional biochemical assay, matrix-assisted laser desorption/ionization time-of-flight mass spectrometry, PCR, and nanoparticle probe technology. Gram staining is a useful tool to guide an empirical therapy before final culture results are available (5). However, the Gram staining method has wide variations in sensitivity and specificity. Conventional biochemical assays are time-consuming and labor-intensive. The other systems require specialized equipment and are not widely available, especially in poor-resource countries.

**Fig 1:**
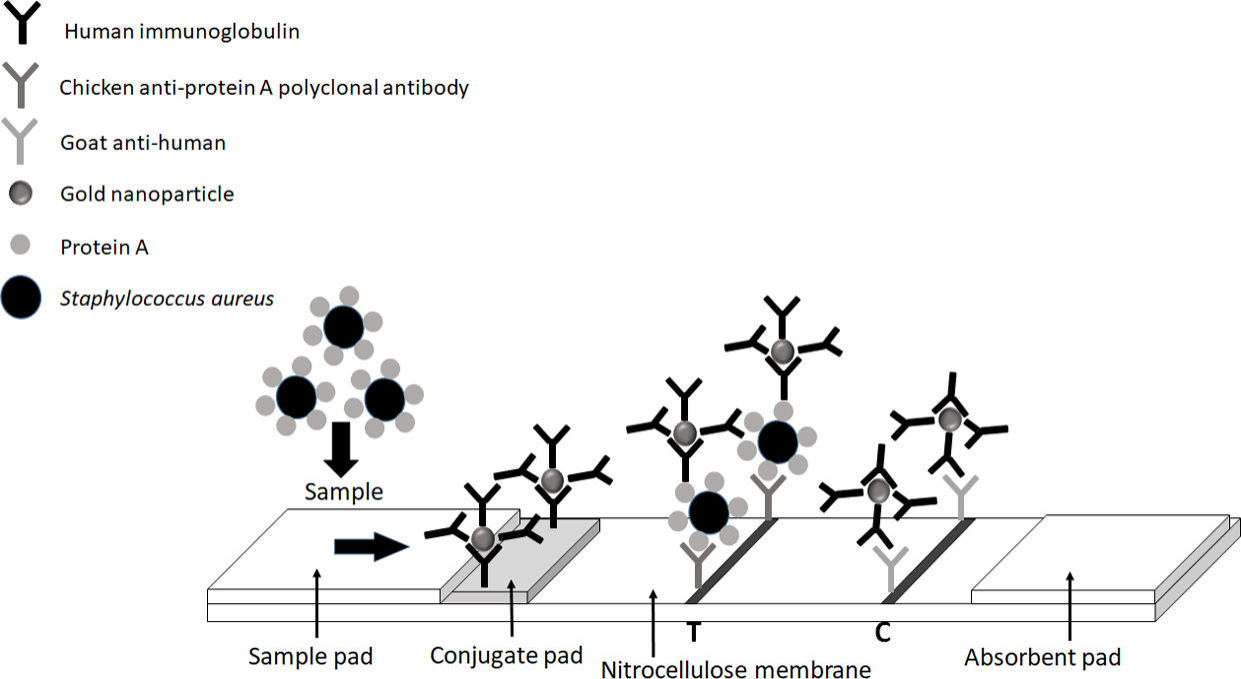
Schematic description of LFIA system: The LFIA consists of the sample pad; the conjugate pad with gold nanoparticle-labeled human immunoglobulin; the nitrocellulose membrane with a test line (T) consisting of chicken anti-protein A polyclonal antibody; a control line (C) consisting of goat anti-human IgG antibody recognizing the gold nanoparticle-labeled human immunoglobulin; and the absorption pad.

A colloidal gold-based lateral flow immunoassay (LFIA) is a rapid, easy-to-use, and inexpensive method. No instrumentation or experts are required. Generally, antibody-based LFAs require the screening process of paired antibodies [conjugate (capture) and detection antibodies] for the sandwich structure reaction, which is usually associated with a high cost of the antibodies. The Fc region of mammalian IgG can naturally conjugate with protein A on the cell surface of *S. aureus* (6). The LFIA technique has been applied to detect *S. aureus* by using a specific antibody (anti-protein A) as a conjugated antibody (7–9). However, human IgG has not yet been used as a conjugated antibody and applied to a fully clinical specimen from hospitals. Therefore, we developed the colloidal gold-based LFIA assay by using a human IgG as a conjugated antibody for the detection of *S. aureus* (Fig. 1), which makes the colloidal gold-based LFIA assay more cost-effective for the routine detection of *S. aureus*. We also evaluated the performance of the test in three hospitals.

## RESULTS

### The detection limit of the LFIA method

**Fig 2:**
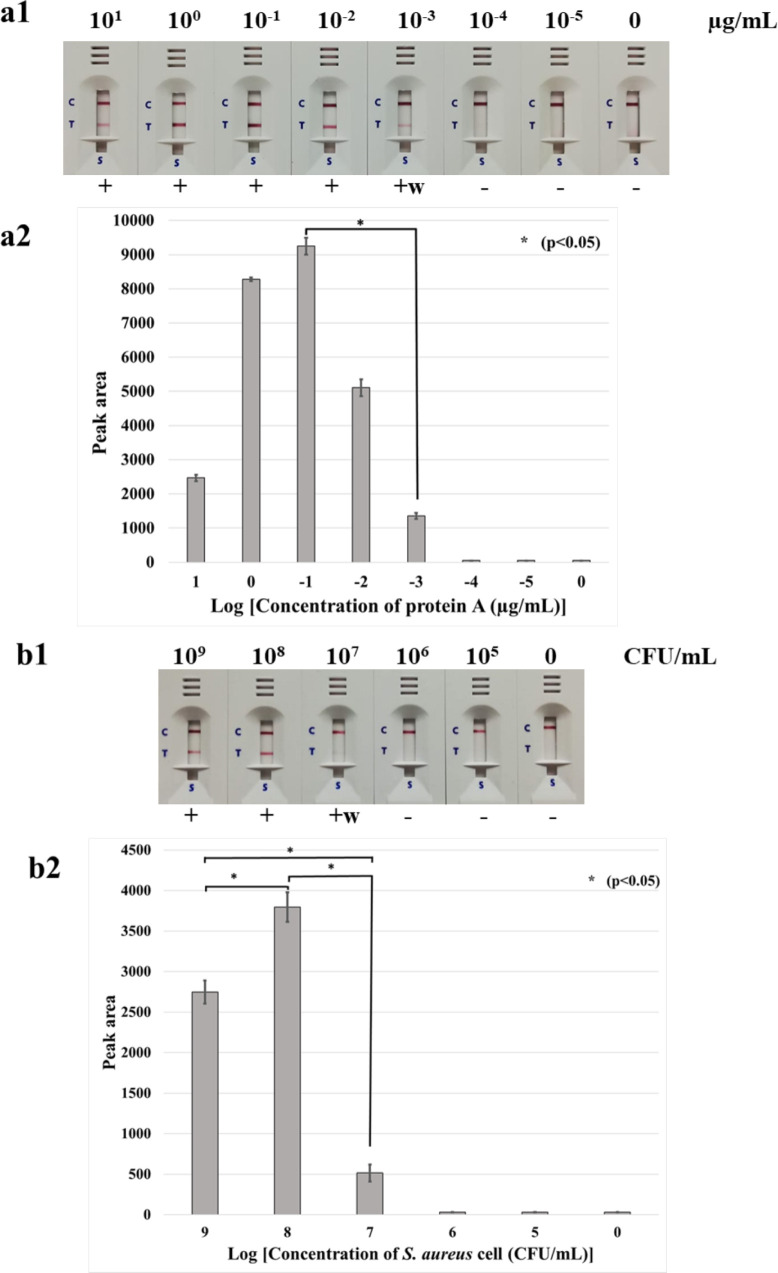
The detection limit of the developed LFIA for detection of protein A in purified protein (10^1^–10^−5^ µg/mL) (a1 and a2) and bacterial colonies (10^9^–10^5^ CFU/mL) (b1 and b2). For the detection limit of purified protein, the concentrations of purified protein A (Arista Biological) varied from 10^1^ to 10^−5^ µg/mL. (a2 and b2) the bar graph representing the peak area of the test line responding from a1 and b1. For the detection limit of bacterial colonies, the bacterial suspension to a turbidity of 1.0 McFarland standard (3 × 10^8^ CFU/mL) was serially 10-fold diluted and confirmed by plating on agar for determination of the CFU. With 10^1^–10^0^ µg/mL of purified protein A and 10^9^ CFU/mL of bacterial colonies, the LFIA showed results due to the “hook” effect. C, control line; T, test line; +, positive result; +w, weakly positive (two red lines of both test and control lines); −, negative result (one red line of the control line only).

The detection limit was tested in triplicate. We found no significant difference between the triplicate tests (*P* = 0.24 and 0.06 for the testing with Protein A and bacterial cells, respectively). The comparison of the peak area of each concentration showed significant difference (*P* < 0.05). The present developed LFIA showed a detection limit of 10^−3^ µg/mL of purified protein A. The limit of the LFIA for the detection of *S. aureus* from the colony was 10^7^ CFU/mL (10^6^ CFU/reaction). A result due to the “hook” effect (10) of the LFIA test was found with 10^1^–10^0^ µg/mL of purified protein A and 10^9^ CFU/mL of bacterial colonies (Fig. 2).

### Detection of *S. aureus* using LFIA in spiked blood culture samples

Using the PCR as a gold standard method, the LFIA strip could detect all *S. aureus* isolates in spiked blood culture samples (sensitivity of 100%; 95% CI: 94.2%–100.0%) within 15 min (no significant difference between the results of the LFIA test and PCR method, *P* = 0.36–0.75 by using Z-test for independent proportion). Weakly positive results were seen in 11 of 79 *S*. *aureus* samples. The test also showed no false positive result (100% specificity; 95% CI: 92.4%–100.0%) (Table 1).

**TABLE 1:** Performance of the LFIA using human immunoglobulin–gold nanoparticle conjugate for detection of *S. aureus* in spiked blood culture samples

Pathogens (*n*)	No. of isolates tested by				
	PCR for *spa* gene		LFIA		
	Positive	Negative	Positive	Weakly positive	Negative
Gram-positive bacteria (126)					
*Staphylococcus aureus* (79)	79	0	68	11	0
*Staphylococcus cohnii* subsp. *urealyticus* (3)	0	3	0	0	3
*Staphylococcus arlettae* (2)	0	2	0	0	2
*Staphylococcus chromogenes* (2)	0	2	0	0	2
*Staphylococcus capitis* (2)	0	2	0	0	2
*Staphylococcus sciuri* (1)	0	1	0	0	1
*Staphylococcus epidermidis* (1)	0	1	0	0	1
*Staphylococcus caprae* (1)	0	1	0	0	1
*Staphylococcus xylosus* (1)	0	1	0	0	1
*Staphylococcus vitulinus* (1)	0	1	0	0	1
*Staphylococcus haemolyticus* (1)	0	1	0	0	1
*Staphylococcus saprophyticus* (1)	0	1	0	0	1
*Staphylococcus* spp. (1)	0	1	0	0	1
*Enterococcus faecalis* (12)	0	12	0	0	12
*Enterococcus faecium* (9)	0	9	0	0	9
*Enterococcus avium* (1)	0	1	0	0	1
*Enterococcus raffinosus* (1)	0	1	0	0	1
*Enterococcus* spp. (1)	0	1	0	0	1
*Streptococcus pyogenes* (2)	0	2	0	0	2
*Streptococcus suis* (2)	0	2	0	0	2
*Bacillus cereus* (1)	0	1	0	0	1
*Listeria* spp. (1)	0	1	0	0	1
Gram-negative bacteria (11)					
*Escherichia coli* (4)	0	4	0	0	4
*Klebsiella pneumoniae* (3)	0	3	0	0	3
*Salmonella* group D (1)	0	1	0	0	1
*Proteus penneri* (1)	0	1	0	0	1
*Citrobacter* spp. (1)	0	1	0	0	1
*Acinetobacter baumannii* (1)	0	1	0	0	1
Yeast (1)					
*Candida albicans* (1)	0	1	0	0	1
**Total (138)**	**79**	**59**	**68**	**11**	**59**

### Evaluation of the LFIA for direct detection in bacterial colonies and positive blood culture bottles in three hospitals

Testing with the bacterial colonies, the LFIA failed to detect 3 of 160 *S. aureus* isolates. The results showed excellent sensitivity and specificity (98.1% and 100%; 95% CI: 94.1%–99.5% and 92.6%–100.0%, respectively). For evaluation in positive blood culture bottles from three hospitals, the LFIA gave 89.7% (95% CI: 79.3%–95.4%) sensitivity, 100% (95% CI: 91.1%–100.0%) specificity compared with those of the PCR method. There were 7 false-negative results from the 68 *S*. *aureus*-positive blood cultures (Table 2).

**TABLE 2:** The performance of the LFIA compared with PCR assay for detecting *S. aureus* in bacterial colonies and positive blood culture samples from three hospitals

Species (*n*)	No. of isolates tested by				
	PCR for *spa* gene		LFIA		
	Positive	Negative	Positive	Weakly positive	Negative
Bacterial colonies (221)	160	61	151	6	64
Hospital A (221)					
Gram-positive bacteria (195)					
*Staphylococcus aureus* (160)	160	0	151	6	3
*Staphylococcus epidermidis* (9)	0	9	0	0	9
*Staphylococcus haemolyticus* (9)	0	9	0	0	9
*Staphylococcus capitis* (2)	0	2	0	0	2
*Staphylococcus simulans* (1)	0	1	0	0	1
*Streptococcus agalactiae* (1)	0	1	0	0	1
*Enterococcus faecalis* (10)	0	10	0	0	10
*Enterococcus faecium* (3)	0	3	0	0	3
Gram-negative bacteria (26)					
*Escherichia coli* (8)	0	8	0	0	8
*Klebsiella pneumoniae* (6)	0	6	0	0	6
*Acinetobacter baumannii* (5)	0	5	0	0	5
*Pseudomonas aeruginosa* (4)	0	4	0	0	4
*Citrobacter* spp. (1)	0	1	0	0	1
*Enterobacter* spp. (1)	0	1	0	0	1
*Stenotrophomonas maltophilia* (1)	0	1	0	0	1
Positive blood cultures (118)	68	50	58	3	57
Hospital A (33)					
Gram-positive bacteria (27)					
*Staphylococcus aureus* (15)	15	0	12	0	3
*Staphylococcus epidermidis* (5)	0	5	0	0	5
*Staphylococcus hominis* (3)	0	3	0	0	3
*Staphylococcus haemolyticus* (1)	0	1	0	0	1
*Staphylococcus capitis* (1)	0	1	0	0	1
*Enterococcus faecalis* (1)	0	1	0	0	1
*Micrococcus luteus* (1)	0	1	0	0	1
Gram-negative bacteria (4)					
*Klebsiella pneumoniae* (2)	0	2	0	0	2
*Acinetobacter baumannii* (2)	0	2	0	0	2
Yeast (1)					
*Candida* spp. (1)	0	1	0	0	1
Mix Gram-positive bacteria and Yeast (1)					
*Staphylococcus epidermidis and Candida parapsilosis* (1)	0	1	0	0	1
Hospital B (76)					
Gram-positive bacteria (65)					
*Staphylococcus aureus* (50)	50	0	44	3	3
*Staphylococcus caprae* (1)	0	1	0	0	1
*Staphylococcus cohnii* (2)	0	2	0	0	2
*Staphylococcus epidermidis* (1)	0	1	0	0	1
*Staphylococcus haemolyticus* (1)	0	1	0	0	1
*Staphylococcus hominis* (2)	0	2	0	0	2
*Staphylococcus warneri* (1)	0	1	0	0	1
*Streptococcus agalactiae* (1)	0	1	0	0	1
*Streptococcus pneumoniae* (1)	0	1	0	0	1
*Enterococcus faecium* (1)	0	1	0	0	1
*Enterococcus faecalis* (1)	0	1	0	0	1
*Micrococcus luteus* (1)	0	1	0	0	1
*Bacillus cereus* (1)	0	1	0	0	1
*Kocuria* spp. (1)	0	1	0	0	1
Gram-negative bacteria (10)					
*Pseudomonas aeruginosa* (2)	0	2	0	0	2
*Escherichia coli* (1)	0	1	0	0	1
*Klebsiella pneumoniae* (1)	0	1	0	0	1
*Klebsiella aerogenes* (1)	0	1	0	0	1
*Salmonella* spp. group D (1)	0	1	0	0	1
*Serratia marcescens* (1)	0	1	0	0	1
*Acinetobacter baumannii* (1)	0	1	0	0	1
*Acinetobacter junii* (1)	0	1	0	0	1
*Burkholderia cepacia* (1)	0	1	0	0	1
Mix Gram-positive and negative bacteria (1)					
*Staphylococcus aureus* and *Proteus mirabilis* (1)	1	0	1	0	0
Hospital C (9)					
Gram-positive bacteria (5)					
*Staphylococcus aureus* (2)	2	0	1	0	1
*Staphylococcus* spp. (2)	0	2	0	0	2
*Staphylococcus haemolyticus* (1)	0	1	0	0	1
Gram-negative bacteria (4)					
*Acinetobacter baumannii* (2)	0	2	0	0	2
*Escherichia coli* (1)	0	1	0	0	1
*Pseudomonas aerugonosa* (1)	0	1	0	0	1

### Stability of the LFIA strip

After storage for 6, 12, and 24 months, the developed LFIA strips still gave clearly positive results with 0.01 µg/mL of protein A without reduced sensitivity but slightly reduced the T-line intensity. The signal intensity stored at 10°C was higher than that of 25°C. These results indicated that the LFIA strips can be stored at 10°C for at least 24 months without losing their efficacy (see Fig. S1 at https://doi.org/10.6084/m9.figshare.24429058).

### Whole-genome sequencing to compare the molecular characteristics of *spa* gene

Genome analysis of one representative with a false-negative result revealed that the *spa* gene is truncated with a stop codon (TAA) in the coding sequences (mutant strain). The *spa* gene was mutated by a novel C to T base substitution (C130T), resulting in a truncated protein A in comparison with the full-length *spa* gene of *S. aureus* N315 (a positive-strain; accession number: BA000018; see Fig. S2 at https://doi.org/10.6084/m9.figshare.24429058).

### Quantitative PCR assay

By the quantitative PCR (qPCR) analysis, the expression of *spa* gene from 2 of the 6 LFIA-weakly positive isolates showed significantly lower levels than those of the LFIA-positive strain (*S. aureus* N315, a wild-type strain; see Fig. S3 at https://doi.org/10.6084/m9.figshare.24429058; *P* = 0.021 and 0.034).

## DISCUSSION

*S. aureus* is a leading cause of bacteremia, infective endocarditis, osteoarticular, skin, and soft tissue, pleuropulmonary, and device-related infections (1). A strong virulence factor called protein A can identify *S. aureus* in clinical specimens (9). Rapid detection and diagnosis of *S. aureus* bacteremia enable prompt and appropriate antibiotic therapy.

In 2020, we developed an LFIA assay using a commercial anti-protein A antibody to detect *S. aureus* in positive blood culture samples which have the sensitivity and specificity of 87.9% and 93.8%, respectively (8). In the present study, we use human immunoglobulin as a conjugate antibody to detect *S. aureus* in hospitals, which has not yet been reported. This modification has several advantages, including a cost reduction because human immunoglobulin may be prepared in-house from the leftover pool-serum samples. Human immunoglobulin preparation is simple, rapid, and no requirement for laboratory-animal. In addition, the detection limit for LFIA using human immunoglobulin as a conjugate antibody was lower than those of previously developed LFIA using commercial anti-protein A antibody (see Fig. S4 at https://doi.org/10.6084/m9.figshare.24429058).

In this study, neither false-positive nor false-negative results were found when tested with spiked blood culture samples. However, while testing in the laboratories of three hospitals, the LFIA strip failed to detect 3 of the 160 and 7 of 68 samples with colonies and positive blood culture samples, respectively. The genome analysis of one representative of the false-negative isolate revealed a mutation in the *spa* gene. This mutation can cause a truncated protein A which may affect the protein A expression or cause impaired function of the protein A (unable to bind with human IgG–gold nanoparticles). Brignoli et al. (11) reported a mutation in the *spa* 5′ UTR sequence affecting the ribosome entry site which is responsible for the loss of protein A expression. In addition, the absence of protein A expression has occurred in geographically distinct strains through several different molecular mechanisms, such as mutation, leading likely to have altered translation, and transcriptional deregulation (11). Therefore, these false-negative results may be due to the low amount or lack of protein A production in some individual strains (12). In addition, *spa* gene expression was determined among six representatives of weakly positive-LFIA isolates by qPCR. It was found that four isolates had low *spa* mRNA levels which may correspond to the low amount of protein A. However, the absence of protein A expression may be associated with other factors, such as higher capsule production (11). Brignoli et al. (11) evaluated the expression of a *spa* in a collection of staphylococcal strains, about 7% of which did not express the *spa* gene, despite the presence of the gene. Several factors or other changes in the virulence gene expression profile have been reported to influence the *spa* expression (11, 13). The regulation of *spa* expression has been found to be complicated which involved multiple factors, including Agr, Rot, ArlR-ArlS, MgrA, SarA, SarS, and SarT (13, 14). It was reported that the upstream promoter sequences were necessary for Agr system regulation of *spa* expression. The SarS and SarA functioned as the activator and repressor of *spa* expression, respectively (13). However, the network of the regulation system is complex and still unclear. Therefore, further study should be performed to gain insights into the reasons for the false-negative results of the LFIA for *S. aureus* detection.

Recently, a silver solution has been reported to enhance the sensitivity of the LFIA test (15–17). We have performed the LFIA test with a silver solution to amplify the signals (see Fig. S5 at DOI: 10.6084/m9.figshare.24429058). However, the reagent was unstable and extremely sensitive to light as suggested by Tsai et al. (9). Moreover, after adding the silver solution, it required an additional 10 min for the final results. Furthermore, there are several factors that affect the signal of LFIA such as the type of buffers and membranes (18). We have investigated the effect of various running buffers, conjugate pads, and nitrocellulose membranes, to select the optimum condition for the best performance of the LFIA strip (8).

For the detection limit of bacterial colonies, the bacterial suspension to a turbidity of 1.0 McFarland standard (~3 × 10^8^ CFU/mL) was serially 10-fold diluted and confirmed by plating on agar for determination of the CFU. However, the McFarland Standard is designed to be used for estimating the concentrations of bacterial cells especially Gram-negative bacteria such as *E. coli*. This estimate becomes uncertain with organisms outside the normal usage as different species of bacteria differ in size and mass. The numbers are valid only for those microorganisms similar to *E. coli* (19). The McFarland method has low accuracy for the estimation of the bacterial populations (20). In this study, the limit of the LFIA for detecting *S. aureus* from bacterial colonies was 10^7^ CFU/mL (10^6^ CFU/reaction). This inoculum could be easily suspended from grown colonies on routine culture plates. In addition, a positive blood culture bottle usually gives a bacterial population of more than 10^7^ CFU/mL. Therefore, the LFIA can be reasonably practical for application in hospitals.

This method has several advantages, including a simple step of sample preparation, extremely easy to use, good performance, rapid, low cost (approximately $2 per test), easy to store, and long shelf life. In addition, special equipment or skilled technicians are not required.

This study provides a preliminary validation of the developed LFIA in three hospitals. Though the number of positive blood culture samples was limited, the results showed that the LFIA can be used to detect *S. aureus* in hospitals. However, a full performance test should be further evaluated in hospitals with a larger sample number.

In conclusion, the developed LFIA exhibits high sensitivity and specificity for detecting *S. aureus* in grown blood culture samples. It could be used as an alternative method for rapid identification of *S. aureus* which contributes to an early diagnosis and facilitates an effective treatment and retards the disease progression. The LFIA may have potential for routine service, particularly in low-resource settings.

## MATERIALS AND METHODS

### Bacterial strains

A total of 138 bacterial isolates, comprising 79 *S*. *aureus* and 59 non-*S*. *aureus*, were collected from Hospitals in Thailand between October 2021 and June 2022 (Table 1). All clinical isolates were stored in skimmed milk with 15% glycerol at −20°C and identified by using standard biochemical tests and PCR techniques. *S. aureus* strain N315 was used as a positive control.

### Preparation of human immunoglobulin

Human immunoglobulin was prepared from the pooled serum of healthy volunteers. It was purified according to the instructions of the HiTrap Protein A HP column (Cytiva, Massachusetts, USA; catalog no. 17040201). After purification, the Vivaspin protein concentrator spin columns (20 and 30 kDa MWCO PES; Cytiva, Massachusetts, USA) were used for the concentration of human immunoglobulin in phosphate buffered saline.

### Creating and assembling the LFIA

### Conjugation of human immunoglobulin antibody and gold nanoparticles

Human immunoglobulin was used to coat the gold nanoparticles. The conjugation process was performed according to the protocol of a previous study (8), except that human immunoglobulin was used as a conjugate antibody with gold nanoparticles (13 nm; Fig. 1). Finally, the labeled conjugate was put onto a conjugation pad using glass fibers (GF33; Whatman Schleicher & Schuell, Dassel, Germany) and dried at 37°C for 2 h, then kept at 4°C for further use.

### Preparation of LFIA strips

Chicken anti-protein A polyclonal antibody (1.0 mg/mL; Arista Biological, Allentown, PA, USA; the purity of protein A > 98% at 214 nm by HPLC) and goat anti-human IgG antibody (1.0 mg/mL; Lampire Biological Laboratories, Pipersville, PA, USA) were dispensed to the nitrocellulose membrane (UniSart CN140; Sartorius Stedim Biotech SA) to be the test and the control lines, respectively, by using a lateral flow dispenser (XYZ3000 Dispensing Platform; BioDot Inc., Irvine, CA, USA; 1 µL/cm) and dried at 37°C for 1 h. The four parts of LFIA including sample pad (cytosep 1660; Pall Gelman Sciences), nitrocellulose membrane [UniSart CN140 NCM (Sartorius Stedim Biotech SA)], conjugate pad (GF33; Whatman Schleicher & Schuell), and absorbent pad (Whatman ABS. PAD #470; Whatman Schleicher & Schuell) were assembled as described previously (8). The treated glass fiber conjugate pad, sample pad, and absorbent pad were assembled to the backing card with a 0.2 cm overlap between each component (Fig. 1). Then the LFIA was cut into 4 mm × 6 cm strips using a CM5000 Guillotine Cutter (BioDot Inc., Irvine, CA, USA). The strips were kept at 4°C in a desiccator.

### Molecular characterization of *S. aureus* by *spa* gene

According to the technique described by Shittu et al. (21), bacterial DNA was extracted using an achromopeptidase enzyme (Sigma-Aldrich, St. Louis, MO, USA). After the 1-min centrifugation, the supernatant was used as a DNA template for the PCR reaction. The protocol and primer for the detection of the *spa* gene were described previously (22). The forward primer of 5′-(1522) TCAAGCACCAAAAGAGGAAGA (1544)–3′ and the reverse primer of 5′-(1784) GTTTAACGACATGTACTCCGTTG (1806)-3′ were used. PCR reaction contained 200 µM of each deoxynucleotide triphosphate, 1 U *Taq* polymerase, 1× PCR buffer, 2 mM MgCl_2_, and 0.5 µM of each primer. The final volume was adjusted to 25 µL with sterile water. PCR amplification was performed with the following conditions: initial denaturation step at 94°C for 5 min; 30 cycles of 94°C for 2 min, 60°C for 1 min, and 72°C for 1 min with a final elongation step at 72°C for 7 min and hold at 4°C. PCR products were detected by electrophoresis in 2.0% agarose gel electrophoresis and stained with 0.5 µg/mL of ethidium bromide. A reference strain of *S. aureus* (NCTC10442) was used as *spa* gene-positive control.

### Detection limit of the LFIA

The concentrations of purified protein A (Arista Biological) that varied from 10^−1^ to 10^−5^ µg/mL were tested with the LFIA strips. The control line and/or test line would appear as a pink-purple line. The presence of both control and test lines indicated a positive result. The presence of only the control line but not the test line indicated a negative result.

To determine the detection limit of the LFIA for bacterial colonies, a 1 µL loop full of bacterial colonies cultured on blood agar (Oxoid, Hampshire, UK) at 35°C for 18 to 24 h was suspended in 1.5 mL microcentrifuge tubes containing 200 µL of distilled water with turbidity of McFarland Standard no. 1.0 (approximately 3 × 10^8^ CFU/mL). Then the bacterial suspension was serially 10-fold diluted from 10^−1^ to 10^−7^, and a bacterial colony count was performed to check the concentration of cells in the suspension. In addition, each bacterial suspension was boiled for 3 min in a heat block at 100°C, and allowed to cool for 2 min, and 100 µL of the 10^0^–10^−5^ dilutions were used as samples. The bacterial solution was dropped into the sample pad of LFIA and added to 100 µL of buffer (Kestrel Bio Sciences, Pathumthani, Thailand). The lowest protein concentration and/or the least colony count that provided a positive result were considered the detection limit.

### LFIA imaging and quantitative analysis of signal

The LFIA was imaged with a smartphone (Huawei Nova 2i, Huawei Base, Shenzhen, China) inbuilt camera at a fixed setting (ISO 50). The smartphone camera was placed at a 90° angle and at a distance of 10 cm above the LFIA. The intensity of the test lines was analyzed using ImageJ software (version 1.53a; National Institute of Health, Bethesda, MD, USA). The intensity of the LFIA signal (test line) was expressed as peak area (23).

### Detection of *S. aureus* from spiked blood culture samples

The spiked blood culture samples of 79 and 59 isolates of *S. aureus* and non-*S*. *aureus,* respectively (Table 1), were used to evaluate the efficacy (sensitivity and specificity) of the LFIA strip for direct detection of *S. aureus*.

**Fig 3:**
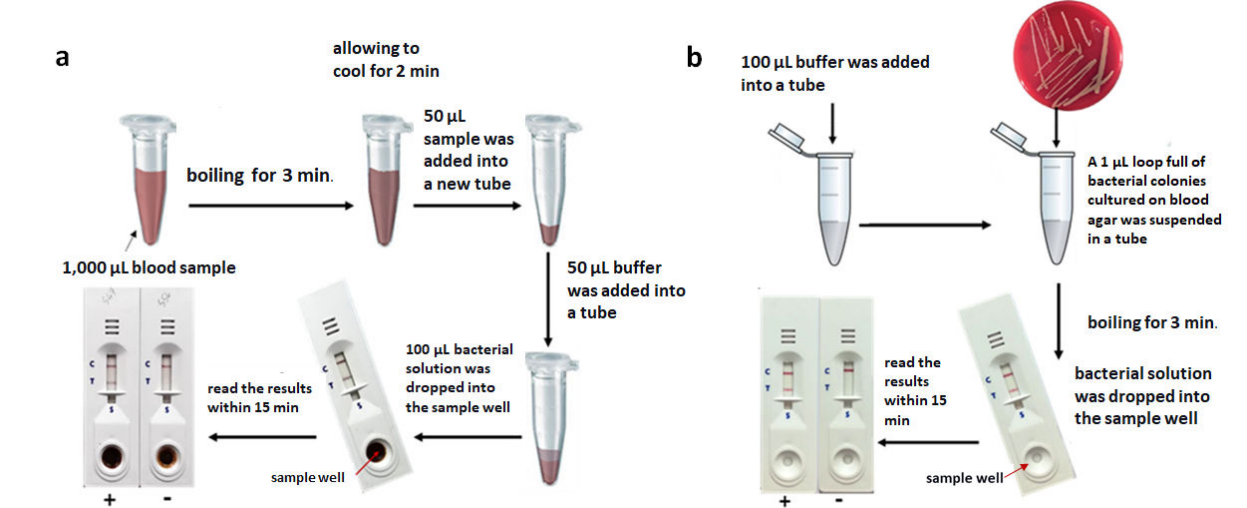
The LFIA protocol for detection of *S. aureus* directly from blood culture samples (**a**) and bacterial colonies (**b**).

The preparation of spiked blood culture samples was performed as described previously (8). Bacterial antigen extraction was accomplished by boiling 1,000 µL of the samples for 3 min in a heat block at 100°C and allowing it to cool for 2 min. Then, 50 µL of the sample was added to 50 µL of buffer (Kestrel Bio Sciences, Pathumthani, Thailand). The total 100 µL of bacterial solution was dropped into the sample pad of LFIA, and the results were read within 15 min with the naked eye (Fig. 3). The results were compared to those of the PCR method, culture, and biochemical tests as the gold standard. In addition, the Staphaurex Plus Latex Agglutination Test (Thermo Scientific., MA, USA) was used to test the isolates with weakly positive and negative results. This test detects both the clumping factor and staphylococcal protein A. Reference strains of *S. aureus* (NCTC10442) were used as a protein A-positive control.

### Evaluation of the LFIA for direct detection in bacterial colonies and positive blood culture bottles from hospitals

The results of the sample were classified in a binary manner (Yes/No) as either positive or negative. The performance of the LFIA test for *S. aureus* detection was blindly evaluated by medical technologists in three hospitals. The LFIA was tested with 221 bacterial colonies and 33 positive blood culture samples in Hospital A. Furthermore, the LFIAs were initially evaluated in 76 and 9 positive blood culture bottles from Hospital B and Hospital C, respectively.

A 1 µL loop full of bacterial colonies cultured overnight on blood agar (Oxoid) at 35°C was suspended in 1.5 mL microcentrifuge tubes containing 100 µL of running buffer (Kestrel Bio Sciences). The suspension was mixed to homogeneity, and boiled for 3 min in a heat block at 100°C, and allowed to cool for 2 min. The suspension was applied to the sample well of an LFIA strip. The result was read within 15 min at room temperature. For the positive blood culture bottles that were flagged by the Microbial Detection System, the samples were tested directly following the protocol for spiked blood cultures, as mentioned above (Fig. 3). The results of the LFIA were compared with the PCR method for *spa* gene, culture and biochemical tests as the gold standard. In addition, weakly positive- and negative-result strains were subjected to the Staphaurex Plus Latex Agglutination Test for confirmation.

### Stability of the LFIA strips

To evaluate the stability of the developed LFIA, the strips were stored under two conditions, at 25°C and 10°C, and tested for their sensitivity and intensity of T-line after the storage at 6, 12, and 24 months.

### qPCR assay for *spa* gene expression analysis

#### RNA extraction and cDNA synthesis

Six isolates of weakly positive-LFIA representatives were selected for analysis by qPCR, compared with *S. aureus* N315, a wild-type strain (LFIA-positive). Bacterial colonies of the selected isolates and *S. aureus* ATCC 25923 strains cultured on blood agar at 37°C for 24 h, were harvested and used for RNA extraction. RNA isolation was performed by the TRIzol method using Trizol reagent (Invitrogen, Waltham, MA, USA) as described by Oo et al. (24). Total RNA concentration was determined using spectrophotometry at 260 nm, and 1 µg total RNA of each sample was subjected to cDNA synthesis using Maxime RT PreMix Kit (iNtRON Biotechnology, Gyeonggi-do, Korea). The total reaction volume was 20 µL per reaction, and the details were generated by following the Maxime RT PreMix Kit protocol. The cDNA was incubated at 45°C for 60 min, followed by RTase inactivation at 95°C for 5 min. cDNA concentration was determined using spectrophotometry at 260 nm, and cDNA was then used for qPCR.

#### *Quantitative real-time* PCR

The real-time PCR for *spa* gene was carried out using 100 ng of cDNA and the primer set as previously described (22). The primers for 16S rRNA (1,478 bp) were as follows: forward, 5′-CCTGGCTCAGGATGAACG-3′and reverse, 5′-AATCATTTGTCCCACCTTCG-3′ (25). The qPCR was performed using SsoAdvanced Universal SYBR Green Supermix (Catalog #1725271, Bio-Rad Laboratories, Hercules, CA) and a real-time PCR instrument (Applied Biosystems QuantStudio 6 Flex Real-Time PCR System). The qPCR conditions were 95°C for 5 min; 40 cycles of 95°C, 15 s; 60°C, 30 s and 72°C, 30 s, respectively. The amplification products were confirmed by a specific melting curve analysis. The relative expression of genes was calculated using the 2^−∆∆Ct^ formula with 16S rRNA as the housekeeping gene, along with *S. aureus* ATCC 25923 growth control.

### Whole-genome sequencing to compare the molecular characteristics of *spa* gene

We investigated the molecular characteristic of the *spa* gene from a representative with LFIA false-negative result by whole-genome sequencing. The DNA was extracted by the phenol-chloroform method (26) and submitted to NovogeneAIT Genomics Singapore for WGS. The DNA sequencing was performed on the NovaSeq 6000 system (Illumina, San Diego, CA) generating 150 bp paired-end reads with >30× coverage. The raw reads were assembled *de novo* and annotated using Unicycler (27) and RAST tool kit (RASTtk) (28) available in the Bacterial and Viral Bioinformatics Resource Center (https://www.bv-brc.org/). Mutations in the *spa* gene were analyzed by multiple sequence alignment using clustalw2 (https://www.ebi.ac.uk/Tools/msa/clustalw2/) compared with the genome of *S. aureus* N315, a wild-type strain (LFIA-positive; accession number: BA000018).

### Statistical analysis

The expression of the *spa* gene from qPCR between the *S. aureus* isolates with LFIA-weakly positive and LFIA-positive was compared using Student’s *t*-test. The results from the LFIA test and PCR method were compared by using the Z-test for independent proportion (Stata analysis version 10.0, College Station, Texas, USA). The sensitivity, specificity, and 95% confidence interval of the LFIA method were calculated using a free software vassarStats (http://vassarstats.net/) (29) using the PCR method as the gold standard. The difference in triplicate peak area of the LFIA was tested by one-way repeated measures analysis of variance (ANOVA). The differences between the peak areas of each concentration were tested using one-way ANOVA analysis. The *P* value of ˂0.05 was regarded as statistically significant.
